# Global Protein Interactome Mapping in Rice Using Barcode‐Indexed PCR Coupled with HiFi Long‐Read Sequencing

**DOI:** 10.1002/advs.202416243

**Published:** 2025-01-22

**Authors:** Xixi Liu, Dandan Xia, Jinjin Luo, Mengyuan Li, Lijuan Chen, Yiting Chen, Jie Huang, Yanan Li, Huayu Xu, Yang Yuan, Yu Cheng, Zhiyong Li, Guanghao Li, Shiyi Wang, Xinyong Liu, Wanning Liu, Fengyong Zhang, Zhichao Liu, Xiaohong Tong, Yuxuan Hou, Yifeng Wang, Jiezheng Ying, Abdullaev Mirtemir Baxodir ugli, Mukhammadjon Arabboevich Ergashev, Sanqiang Zhang, Wenya Yuan, Dawei Xue, Jianwei Zhang, Jian Zhang

**Affiliations:** ^1^ State Key Lab of Rice Biology and Breeding China National Rice Research Institute Hangzhou 311400 China; ^2^ National Key Laboratory of Crop Genetic Improvement Hubei Hongshan Laboratory Huazhong Agricultural University Wuhan 430070 China; ^3^ Rice Research Institute of Uzbekistan Tashkent 111506 Uzbekistan; ^4^ Hubei Agricultural Machinery Engineering Research and Design Institute Hubei University of Technology Wuhan 430068 China; ^5^ State Key Laboratory of Biocatalysis and Enzyme Engineering School of Life Sciences Hubei University Wuhan 430062 China; ^6^ College of Life and Environmental Sciences Hangzhou Normal University Hangzhou 311121 China

**Keywords:** barcode‐indexed PCR, HiFi sequencing, protein–protein interactome, rice

## Abstract

Establishing the protein–protein interaction network sheds light on functional genomics studies by providing insights from known counterparts. However, the rice interactome has barely been studied due to the lack of massive, reliable, and cost‐effective methodologies. Here, the development of a barcode‐indexed PCR coupled with HiFi long‐read sequencing pipeline (BIP‐seq) is reported for high throughput Protein Protein Interaction (PPI)identification. BIP‐seq is essentially built on the integration of library versus library Y2H mating strategy to facilitate the efficient acquisition of random PPI colonies, semi‐mechanized dual barcode‐indexed yeast colony PCR for the large‐scale indexed amplification of bait and prey cDNAs, and massive pac‐bio sequencing of PCR amplicon pools. It is demonstrated that BIP‐seq could map over 15 000 high‐confidence (≈62.5% could be verified by Bimolecular fluorescence Complementation (BiFC)) rice PPIs within 2 months, outperforming the other reported methods. In addition, the obtained 23 032 rice PPIs, including 22,665 newly identified PPIs, greatly expanded the current rice PPI dataset, provided a comprehensive overview of the rice PPIs networks, and could be a valuable asset in facilitating functional genomics research in rice.

## Graphical abstract

BIP‐seq is developed, a cost‐effective pipeline for high‐throughput protein–protein interaction (PPI) identification using yeast two‐hybrid system. BIP‐seq successfully map over 15 000 high‐confidence rice PPIs (≈62.5% could be verified by BiFC) within 2 months. A rice PPI database (RiPPID) containing 23032 PPIs is established, which greatly enhances the understanding of the interactome in rice.

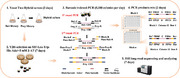

## 1 Introduction

Proteins execute nearly all biological functions through protein–protein interactions (PPIs). A protein‐protein interactome (PPIome) is defined as the complete collection of all physical PPIs within a cell.^[^ ^1^ ^]^ Since the invention of yeast two‐hybrid (Y2H), the first technique for PPI detection in 1989,^[^ ^2^ ^]^ PPI has become a critical part of the current biological research, which dramatically facilitated the establishment of important, reliable biological models.^[^ ^3^ ^]^ In addition to the Y2H, a series of other PPI detection techniques such as bacterium two‐hybrid, split ubiquitin system, bimolecular fluorescence complementation (BiFC), fluorescence resonance energy transfer (FRET), affinity purification followed by MS (AP‐MS) and proximity labeling followed by MS (PL‐MS) have been developed in the past four decades.^[^ ^4^, ^5^, ^6^ ^]^ Nevertheless, attaining PPIome coverage for each species is arduous, mainly due to the scarcity of reliable, cost‐effective methods.

Early efforts in PPIome mapping relied on the manual construction of open reading frames collections (ORFeomes) and pairwise test of the ORFeomes using Y2H, which have profiled thousands of PPIs in worms, human, yeast and Arabidopsis, respectively.^[^ ^7^, ^8^, ^9^, ^10^, ^11^, ^12^ ^]^ A “Stitch‐Seq” method that combines PCR amplicon stitching with next‐generation sequencing was reported to enable the identification of 979 high‐quality human PPIs from 5200 Y2H colonies.^[^ ^13^ ^]^ Cre‐reporter‐mediated Y2H coupled with next‐generation sequencing (CrY2H‐seq) and recombination‐based “library versus library” Y2H system (RLL‐Y2H seq) pipelines both are derived from modified Y2H system using a Cre recombinase interaction reporter. The coding sequences of the interactive proteins in the systems could be intracellularly fused by Cre recombinase and directly PCR amplified for next‐generation sequencing.^[^ ^14^, ^15^ ^]^

In 2015, the human PPIome dataset was greatly expanded using an AP‐MS strategy. Two independent groups fused thousands of the human ORF clones with FLAG‐ or GFP‐ epitope tags and expressed the recombinant proteins in specific cell lines for AP‐MS, and finally obtained 23744 and 28500 human PPI datasets, respectively.^[^ ^16^, ^17^ ^]^ Using a co‐fractionation mass spectrometry (CF‐MS) approach, Mcwhite et al. (2020) chromatographically separated native protein extracts from 13 plant species spanning 1.1 billion years of green plant evolution and developed a pan‐plant protein complex map covering 2 million PPIs.^[^ ^18^ ^]^ Despite its high throughput in PPIome mapping, large‐scale MS‐based approaches are costly and time‐consuming, and the detected PPIs might not be physical.

Here, we report the development of a pipeline called BIP‐seq (Barcode‐Indexed PCR coupled with HiFi long‐read sequencing) for massive, reliable, and cost‐effective PPI detection. BIP‐seq starts from the co‐transformation or mating of the mixed pools of baits and preys for PPI selection on a nutrient‐deficient medium. For each PPI colony, two rounds of PCR amplifications are performed to attach four unique barcodes on the end of the bait and prey cDNA amplicons, enabling the massively multiplexed PPI screening in 10^5^ levels by HiFi long‐read sequencing within five weeks. Employing the BIP‐seq pipeline, we successfully captured over 15785 high‐confidence rice (*Oryza sativa* L.) binary PPIs from 60000 Y2H colonies with a verification rate of 62.5% (25/40) by BiFC, which has expanded the rice PPI dataset eight times larger than before.

## 2 Experimental Section

### 2.1 cDNA Library Construction and Library versus Library Y2H Mating

The normalized bait and prey Y2H cDNA libraries were established through a commercial service by Takara Bio Inc (Dalian, China). Briefly, mRNA was isolated from the ten tissues (root, stem, leaf, panicle, callus, seedling, and seed at 0, 3, 7, 15 DAP) and reverse transcribed using SMART cDNA library construction kit (Cat No. 634901, Clontech, Dalian, China), and directionally cloned into pGADT7 and pGBKT7 vectors, respectively, through the *SfiI* site.

To identify positive wise‐pairs by high‐throughput mating, 0.8 mL of the bait and prey cDNA library stocks in Y187 and Y2HGold strains, respectively, were mixed in a sterilized 2 L flask with 50 mL 2 x YPDA medium, then incubated at 30 °C for 20 h under shaking at 50 rpm. Mated zygote cells were collected and resuspended in 15 mL 0.9% NaCl solution, cultured on nutrient‐deficient medium SD/‐Leu‐Trp‐His‐Ade/+5 mm 3‐AT at 30 °C for 7 d. Yeast colonies bigger than 1–2 mm in diameter were considered to contain positive PPIs.

To exclude the potential PPIs with autoactivities, the bait and prey cDNA libraries were mated with yeast strains containing pGAD‐T7 and pGBK‐T7, respectively. Then, the stock containing over 1 million mated zygotes was selected on SD/‐Leu‐Trp‐His‐Ade/+5 mm 3‐AT at 30 °C for 7 d. All the grown‐out colonies were picked for PCR and HiFi long‐read sequencing identification.

### 2.2 Barcode‐Indexed PCR Using Thermal Cycling Station (TCS)

Single PPI yeast colonies were picked by a high‐throughput microbial colony picker system (QPix 420, Molecular Devices, San Jose, USA) or manually into the wells of a 96‐well plate containing freshly prepared 10 µL 1 x KBB lysis buffer (20 mm KOH, 0.5% BSA, 1 m betaine). Heat treated at 100 °C for 5 min. For the 1st round of barcode‐indexed PCR, a 20.4 µL reaction system containing 10 µL 2 × Phusion Mix, 0.1 µL 10 µm pGADT7 F/R and pGBKT7 F/R, and 10 µL yeast lysate in KBB lysis buffer were applied for PCR in the TCS station. Subsequently, 1 µL of the 1st round PCR product was preceded to the 2nd round PCR system containing 10 µL 2 × Phusion Mix, 0.2 µL 10 µm Block F/R, 0.2 µL 0.33 µm pGADT7 plate F/R and pGBKT7 plate F/R, and 10 µL yeast lysate in KBB lysis buffer were applied for PCR in the TCS station. Finally, tens of thousands of the 2nd round PCR products were collected, column purified, and quantified by Qubit Fluorometer (Novogene, Beijing, China) for HiFi long‐read sequencing. The sequence of the primers and barcodes can be found in Tables S17 and S18 (Supporting Information).

The TCS system consists a six‐axis robot (model: BRTIRUS 15 10A, BORUNTE, Dongguan, China), three water baths (model: DKB‐8, JINGHONG, Shanghai, China), an iron netcase (40 cm × 35 cm × 8 cm) with a maximum loading capacity of 84 standard PCR plates, and a semi‐automatic plate sealer (Miulab, Hangzhou, China). The six‐axis robot runs following the below procedure and coordinates are given in **Table** **1**.

**Table 1:** The process of TCS system.

Step	Temperature and time	Robot arm coordinates
Denaturing	95 °C, 60 S	X, 556.856; Y, ‐440.196; Z, 1293.015; U, ‐177.397, V, 0.377; W, 91
Annealing	55 °C, 75 S	X, 556.860; Y, ‐440.213; Z, 1048.552; U, ‐177.386, V, 0.375; W, 91
Amplification	68 °C, 90 S	X, 533.996; Y, ‐0.093; Z, 1042.137; U, ‐177.285, V, 0.446; W, 91
27 cycles		

Note: 1. Before moving to the next step, the loading case is hanging in the air for 2 s to remove the water in the case. 2. Transferring from one water bath to another takes 2 s.

### 2.3 Yeast Colony Lysis

Three other yeast colony lysis methods were tested in this study to compare the efficiency with KBB lysis buffer. For the SDS lysis, yeast colonies were treated with 30 µL 0.2% (W/V) SDS at 95 °C for 5 min. After a brief centrifuge, 0.2 µL supernatant was aliquoted out as a template for PCR. The microwave lysis method was conducted by heating the yeast colonies in 10 µL water in a microwave (Power 800 W, NN‐ST65JM, Panasonic, Japan) for 5 min, and 0.2 µL of the supernatant was used as the template. For the zymolyase lysis, a mixture of 45 µL sorbitol buffer and 10 µL zymolyase (Cat No. R1020, Solarbio, Beijing, China) was used to lysis the yeast at 37 °C for 2 h. Then, the lysate was 5 times diluted by ddH_2_O, and 1 µL supernatant was preceded for PCR reaction.

### 2.4 HiFi Long‐Read Sequencing and Sequence Demultiplex

HiFi long‐read sequencing was performed by Novogene ltd, Beijing, China. Briefly, PCR products were repaired for Blunt‐End‐Ligation using Template Prep Kit 1.0 – SPv3 (Cat No. 100‐222‐300, Pacific Biosciences, California, USA) and filtered for DNA >15 kb in size using Diagenode Megaruptor system (Cat No. B06010003, Diagenode, Belgium). Then, the filtered DNA was purified to produce the SMRTbell templates using AMPure PB Beads (Cat No. 102‐182‐500, Pacific Biosciences, California, USA), and treated with *Exo*III (Cat No. M0206 V, NEB, Beijing, China) and *Exo*VII (Cat No. M0379S, NEB, Beijing, China) to remove single strand DNA and DNA residues at 37 °C for 1 h. After DNA purification and concentration by AMPure PB Beads and quantification by Agilent DNA 12 000 Kits (Cat No. 5067‐1508RUO, Agilent, Beijing, China), sequencing barcodes were attached to the ends of the SMRTbell templates using polymerase from Binding Kit. Finally, the library was sequenced in SMRT Cells (Cat No. 102‐202‐200, Pacific Biosciences, CA, USA) using DNA Sequencing Reagent Kit (Cat No. 101‐597‐900, Pacific Biosciences, CA, USA).

The raw sequencing data were processed into circular consensus sequences (CCS) using SMRT Link software with parameters “–skip‐polish –min‐passes 1 –min‐rp 0.98.” Then, CCS reads in FASTA format were sequentially aligned to the pGADT7 and pGBKT7 vector backbone sequences (minimum match: 29 bp, maximum mismatch and gap: 6 bp). CCS reads having backbone sequence were further proceeded to align with the fused sequence of block barcode + bridge sequences (matched length: 32–36 bp) and bridge sequences + plate barcode (matched length: 24–28 bp) using “blastn” with parameters “‐task blastn‐short ‐outfmt 6 (maximum mismatch and gap: 2 bp)”, respectively. The qualified reads with identifiable barcodes were preceded to align to the CDS sequences of Nipponbare (*Oryza sativa* L. ssp. *japonica*, Rice genome annotation project version 7.0, http://rice.uga.edu/) using “blastn” with parameters “‐task blastn‐short ‐outfmt 6,” and to determine the locus ID of protein with the highest score value. To determine the ORF fusion with GAL4 domain, the clean reads without vector backbone sequences were aligned with rice protein sequences using “blastx,” and the hits with frame of ± 1 were defined as GAL4‐fused.

### 2.5 Bioinformatics and Statistical Analysis

Cytoscape and Gephi were used to visualize and analyze the network. Network Analyzer and cytoHubba in Cytoscape was used to analyze the degree and hub genes. The data analysis in Gephi was performed to analyze the degree, weight degree, eccentricity, closeness centrality, harmonic closeness centrality and betweenness centrality. The domain data in rice was downloaded in CDD: https://ftp.ncbi.nih.gov/pub/mmdb/cdd/cdd.tar.gz. The cellular localization was downloaded in website: https://wolfpsort.hgc.jp. Variables were expressed as mean± SD. For normally distributed data sets with equal variances, one‐way ANOVA testing followed by Tukey’ test was carried out across groups. Significance was defined as α = 0.05. Statistical analysis was carried out using GraphPad Prism software.

### 2.6 Bimolecular Fluorescence Complementation (BiFC) Assays

To validate the PPIs identified by BIP‐seq, 98 PPIs were randomly picked for BiFC verification. In brief, the CDS of each bait and prey genes were cloned into pCAMBIA1300‐35S‐N‐YFPC and pCAMBIA1300‐35S‐N‐YFPN vectors, respectively, using ClonExpress II One Step Cloning kit (Cat No. C112‐01. Clontech, Dalian, China). The recombinant constructs were transformed into Agrobacterium GV3101, and infiltrated into the leaf epidermal cells of 3 week old *Nicotiana benthamiana*. After 36 h of incubation, fluorescence signals were observed under a Zeiss LSM710 confocal laser‐scanning microscopy (Carl Zeiss AG, Jena, Germany). Sequence of the primers can be found in Tables S17 and S18 (Supporting Information).

### 2.7 Y2H Assays

Y2H assays were performed using the match‐marker GAL4 two‐hybrid system from Clontech, CA, USA, following their specified protocol. The coding sequence of 116 genes was amplified from the cDNA of variety Nipponbare and cloned into pGADT7 and pGBKT7 vectors. The primers used are listed in Table S17 (Supporting Information). The constructs were co‐transformed into the yeast strain Y2H Gold. The yeast strains were selected on SD/‐Trp‐Leu and SD/‐Trp‐Leu‐His‐Ade medium for 4 days at 30 °C.

### 2.8 Phenotypic Analysis of the Genetic Materials

The *nac23* and *bzip58* mutants were generated by a reported CRISPR‐cas9 system. Briefly, the gDNA sequences that annealed 19 bp genomic DNA were ligated into the pYLCRISPR/cas9‐MH vector using *Bsa*I site. All binary vectors were transformed into Nipponbare and Dongjin backgrounds via the Agrobacterium‐mediated transformation method. All plants were grown in the experimental field of China National Rice Research Institution in Hangzhou, China. For the cold stress treatment, 7 d old rice seedlings were transferred into a growth chamber for 5 days (4 °C, 14 h: 10 h, light: dark, 200 µmol m^−2^ s^−1^ photon density), and restored under 28 °C for 7 d to count the survival rate.

## 3 Results

### 3.1 BIP‐Seq Workflow

**Figure 1:**
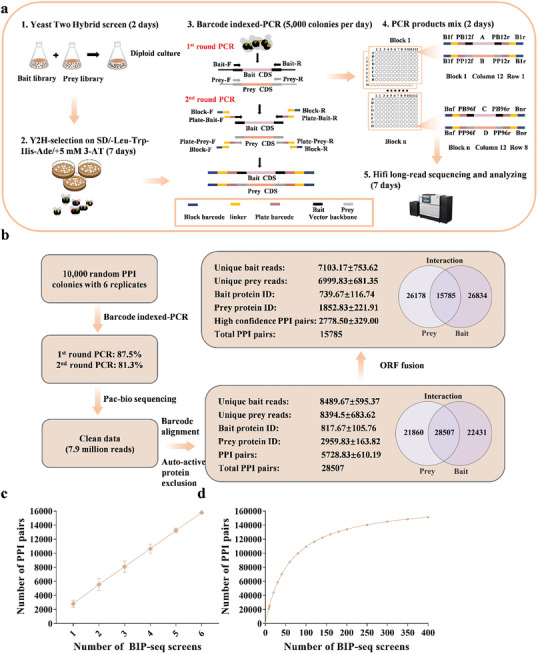
The workflow and bioinformatic analysis process of BIP‐seq. a) The workflow of BIP‐seq technology. b) The bioinformatic analysis pipeline and output of BIP‐seq results on 60000 random PPI colonies. c) PPI detection efficiency of the BIP‐seq sample size. Each BIP‐seq screen contains 10000 random PPI colonies. Error bars, s.d. d) A Michaelis‐Menton mode‐based curve of PPI detection using BIP‐seq.

We developed a high‐throughput Y2H‐based pipeline to identify PPIs in a given species. As shown in **Figure** **1**a, the methodology begins with the mating of bait and prey cDNA libraries using the Y2H system to obtain random PPI colonies on selective mediums (Figure 1a). Subsequently, a two‐round, dual barcode‐indexed PCR is performed to amplify the bait and prey cDNA fragments from each PPI yeast colony (Figure 1a). For the 1^st^ round PCR, both bait and prey cDNAs from the same colony are simultaneously amplified using the corresponding universe vector backbone primers to enrich the target fragments (Figure 1a).

The 2^nd^ round PCR is conducted using the 1^st^ round PCR products as templates. Three pairs of primers: plate‐bait barcode primers, plate‐prey barcode primers, and block barcode primers were designed in this step. Plate‐bait and plate‐prey barcode primers contain a linker sequence, an 8‐nt plate barcode, and a nest primer annealing to the vector backbone (Figure 1a). The combination of the plate barcode on both ends indicates the coordinate of the amplified bait or prey cDNA within a 96‐well plate. Block barcode primers comprise a 12‐nt block barcode and the same linker sequence as on the plate barcode primers. Bait or prey cDNAs from the same 96‐well plate are amplified using the same block barcode primers, allowing easy tracking of the plate of origin. In the PCR reaction system, plate bait and prey barcode primers were diluted to 1:30 of the amount of the block barcode primers, hence ensuring the attachment of block barcodes on most of the final PCR products (Figure 1a).

Under this system, every PCR sample could be distinguished from each other by the joint identity of vector backbone sequence (bait or prey), plate barcodes (coordinate within a 96‐well plate), and block barcodes (origin of the 96‐well plate) (Figure 1a). Finally, tens of thousands of the amplified bait and prey cDNA fragments are mixed and applied for HiFi sequencing (Figure 1a). Technically, bait and prey cDNA fragments carrying identical barcodes on the ends are derived from the same yeast colony and can thus be defined as PPI pairs. In the meantime, each cDNA library is mated with the corresponding empty vector to obtain the bait or prey proteins with auto‐activities, thus being excluded from the identified PPI pairs.

### 3.2 Optimization of the BIP‐Seq Technology

Direct PCR amplification on yeast colonies is technically challenging due to the rigid poly‐saccharide cell walls of the yeast, particularly on a large scale. Several yeast colony PCR systems, such as SDS lysis, zymolyase lysis, and microwave heat, have been reported, while most methods are either unstable or costly. To this end, we developed a highly effective yeast KBB lysis buffer (20 mm KOH, 0.5% BSA, 1 m betaine). The alkaline KBB solution under high temperature enables fast degradation of the yeast cell wall and release of the plasmids. At the same time, BSA and betaine effectively protected the DNA polymerase from the inhibitors, thus yielding a significantly stable and higher amplification rate than the routinely used SDS lysis and microwave heat methods (Figure S1, Supporting Information). Meanwhile, KBB lysis outperforms zymolyase lysis in processing time and cost, though the PCR yield is similar (Figure S1, Supporting Information).

BIP‐seq relies on the PCR amplification of numerous samples, which requires high throughput thermal cycler systems. To suffice this demand, we invented a simple yet highly efficient thermal cycling station (TCS) (Supplemental file 1). The TCS comprises three water bathes with set temperatures for the PCR denaturing, annealing, and amplification steps, respectively, and a programmed industry robotic arm to move the PCR samples among the three steps. All the 96‐well or 384‐well plates are heat‐sealed with plastic films to prevent the samples from the water; thus, they could be stacked and wholly immersed into the water during the thermal cycling to make full use of the vertical spaces. The capacity of TCS could be flexibly adjusted by the sizes of the water baths. For example, TCS with regular water baths in size of 50 × 30 × 20 cm could simultaneously accommodate up to 85 standard PCR plates each time, significantly improving the PCR throughput (File S1, Supporting Information). The estimated cost for constructing a TCS (approximately 5000 US dollars) is almost equivalent to that of a single commercial thermal cycler, which is affordable to most small molecular biology laboratories worldwide.

During the pre‐test of BIP‐seq, we noticed that the plate or block barcodes of some reads were unrecognizable due to sequence errors, which significantly impaired the identification of the reads. Generally, single usage of each barcode primer needs a larger amount of oligo synthesis. However, it gives a higher identification rate since the barcode from either the 5′ or 3′ end could track the coordinates of the reads. In contrast, when reusing barcode primers, the identification rate may significantly decrease as each read requires recognition of the barcode from both the 5′ and 3′ ends. Nonetheless, this approach offers the advantage of reduced oligo synthesis cost. We designed three strategies for barcode usage during barcode‐indexed PCR (Figure S2, Supporting Information). To distinguish 40 000 independent colonies, strategies I, II, and III require 1202, 498, and 110 pieces of primers, respectively. By analyzing 10 000 randomly selected PCR reads with three replicates, we revealed that strategy I (single use of block and plate barcode primers) tracked the coordinates of 91.94 ± 0.46% and 80.73 ± 0.96% of the bait and prey reads, respectively, and finally 79.98 ± 5.60% of the reads could find a PPI counterpart. Meanwhile, the PPI identification efficiency was only 30.21 ± 2.35% and 14.66 ± 1.37% for strategies II and III, respectively (Figure S2, Supporting Information). Given the significantly higher PPI identification of strategy I, we decided to carry out the barcode‐indexed PCR with a single use of all the barcode primers.

### 3.3 Capability Test of BIP‐Seq on Rice PPI Identification

We tested the power of BIP‐seq on rice with 6 replicates, each containing 10 000 random PPI colonies. Barcode‐indexed PCR results showed 87.5% and 81.3% amplification rates at 1^st^ and 2^nd^ round PCR, respectively (Figure 1b and Figure S3, Supporting Information). HiFi long‐read sequencing of the mixed 60000 PCR products generated a total of 7.9 million reads for barcode alignment. After removing the redundancy, 8489.67 ± 595.37 indexed bait reads and 8394.5 ± 683.62 indexed prey reads could be identified from each replicate, respectively. Subsequently, BLAST analysis matched the reads to 817.67 ± 105.76 bait proteins and 2959.83 ± 163.82 prey proteins, respectively. After removing the PPIs with auto‐activities (Table S1, Supporting Information), 5728.83 ± 610.19 PPI pairs from every 10 000 colonies, and a total of 28507 non‐redundant PPI pairs were finally yielded (Figure 1b).

Given that the bait and prey cDNAs could be open reading frame shifted in the Y2H library, we further analyzed the ORF fusion of bait and prey cDNAs with the GAL4 domains. As a result, 7103.17 ± 753.62 indexed bait reads, and 6999.83 ± 681.35 indexed prey reads covering 739.67 ± 116.74 bait proteins and 1852.83 ± 221.91 prey proteins were identified, respectively. In total, we identified 15785 high‐confidence PPI pairs with an average of 2778.50 ± 329.00 PPIs from each replicate. The results demonstrated the high efficiency of BIP‐seq, as close to 30% of the colonies could be identified as high‐confidence PPIs (ORF fused) (Figure 1b). During our investigation, we have observed that the identified PPI exhibits a linear increase with the replicates (Figure 1c). This finding strongly suggests that the 6 BIP‐seq screens are far from saturating the rice PPIome. As per the Michaelis‐Menton modeled curve, it is estimated that over 400 BIP‐seq screens will be required to achieve a saturated detection of 151307 PPIs in this particular case (Figure 1d).

### 3.4 Rice PPI Dataset

**Figure 2:**
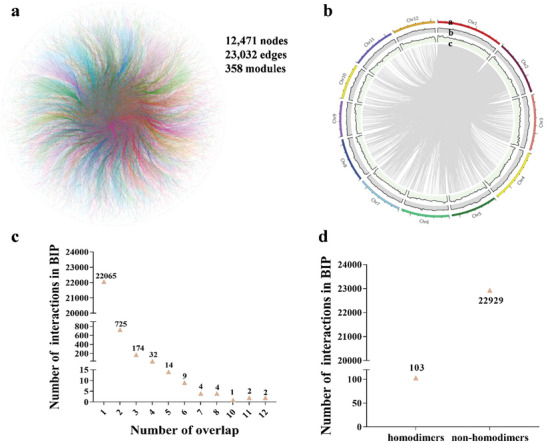
The detailed information of RiPPID. a) The overall picture of RiPPID. The nodes represent proteins in RiPPID; the edges indicate PPI relationships; the modules indicate the nodes were classified in RiPPID with community detection. The various colors represent different modules. b) The distribution of PPI proteins on rice chromosomes. a. chromosomes. b. GC contents. c. gene densities. c) The detection frequencies of PPIs in RiPPID. d) The number of homodimers and non‐homodimers in RiPPID.

**Figure 3:**
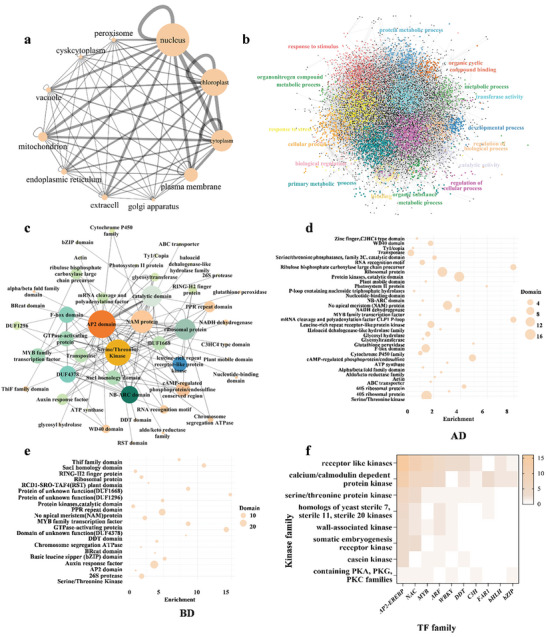
The cellular localization, gene ontology and conserved domain analysis in RiPPID. a) Cellular localization interaction network. Nodes represent cellular components; edges indicate interactions among proteins localized in the corresponding cellular compartments. b) GO analysis of RiPPID in terms of molecular function and biological process. Colored nodes indicate community types enriched in GO annotations. c) The map of interactions between TFs and protein kinases. d,e) The domain enrichment analysis in Bait (BD) and Prey (AD) proteins. The size of the dot indicates the number of proteins within each category. f) The interaction network among conserved protein domains. Nodes represent the conserved domain types, and edges indicate interactions. Only domain pairs containing over 6 PPIs were displayed.

Combining the PPIs obtained above and from other trial tests, we finally profiled 23032 high confidence PPI edges harboring 12741 nodes, termed as Rice‐PPI dataset (RiPPID) (**Figure** **2**a, Table S2, Supporting Information). Compared to the previously reported rice PPI dataset from BioGRID, LCI, Cell 2020, and Rice Net 2.0, RiPPID newly identified 22665 PPIs, representing the largest experiment‐based rice PPI dataset so far (Table S3, Supporting Information). Notably, only a small percentage of RiPPID overlaps with the existing PPI dataset, likely due to the extensive scale of the rice protein interactome and the insufficient coverage of the current data. The PPI nodes are distributed on all the 12 rice chromosomes (Figure 2b). 22065, 725, and 174 PPIs were detected for one, two, and three times, respectively. In contrast, less than 70 PPIs were identified over four times, suggesting that more BIP‐seq screens may be necessary to saturate the rice PPIome (Figure 2c). In addition, 103 proteins exhibit the ability to self‐bind and form homodimers (Figure 2d and Table S4, Supporting Information). A subcellular localization analysis revealed that RiPPID proteins are mainly located in the nucleus, chloroplast, cytoplasm, and plasma membrane (**Figure** **3**a). The PPI could occur within the same cellular compartment or inter‐cellular, which forms a complicated localization‐based network of connections (Figure 3a and Table S5, Supporting Information).

To explore potential interaction models among biological processes and molecular functions, we performed an analysis utilizing Gene Ontology (GO). Our examination of the RiPPID dataset identified 16 distinct communities comprising more than 674 genes, represented as green dots. The analysis revealed that the most significantly enriched GO terms in molecular function were “cellular process” and “metabolic process.” In contrast, the most enriched categories in biological function were “organic cyclic compound binding” and “catalytic activity” (refer to Figure 3b and Tables S6 and S7, Supporting Information). The network analysis illustrated various potential interaction models, including the association of the “cellular macromolecule metabolic process” with the “metabolic process,” the relationship between “binding” and “catalytic activity,” and the connection between “biological regulation” and “response to stimulus.”

In terms of PPIs between protein families with conserved domains, we identified 120 types of inter family PPIs with at least 6 counts, among which AP2‐serine/threonine kinase and AP2‐catalytic domain were highlighted as the most abundant ones (Figure 3c and Table S8, Supporting Information). Meanwhile, protein domains in the AD were enriched in mRNA cleavage factor and ribulose precursor, whereas domains in the BD were mainly enriched in *sacI* homology domain, DUF1296, DUF4378, and GTPase‐activating protein (Figure 3d,e). We further investigated the 120 particular PPIs between protein kinases and transcription factors (TFs) (Figure 3f and Table S9, Supporting Information).

Among the combinations between 8 types of protein kinases and 16 types of TFs, the most abundant interactions were involved in AP2‐EREBP with receptor like kinases or calcium/calmodulin dependent protein kinase (Table S10, Supporting Information). In addition, the count of serine/threonine protein kinases interaction with AP2‐EREP or NAC family is 6 and 4, respectively (Table S10, Supporting Information).

Finally, we randomly picked 40 PPIs to evaluate the reliability of RiPPID in planta using BiFC technology. As a result, 25 out of the 40 PPI pairs were confirmed in tobacco epidermal cells, accounting for a positive rate of 62.5% (Figure S4 and Table S11, Supporting Information). To assess the correlation between BiFC and Y2H, two additional controls were conducted. In the negative control group containing 28 Y2H‐validated negative PPIs (Figure S5a and Table S12, Supporting Information), only one PPI turned out to be positive by BiFC assays, demonstrating a false‐positive rate of 3.6% (Figure S6 and Table S13, Supporting Information). In contrast, three out of the 28 Y2H‐validated positive PPIs (Figure S5b and Table S12, Supporting Information) were found to be negative by BiFC, representing a false‐negative rate of 10.7% (Table S13, Supporting Information). These data strongly suggested that RiPPID is highly reliable.

### 3.5 RiPPID Indicates the Biological Functions of the Interactive Protein Genes

A trait ontology analysis of the RiPPID revealed over 358 PPI modules potentially involving plant height, leaf color, heading date, and other biological processes (Figure S7 and Tables S14 and S15, Supporting Information).

These modules provided an in‐depth view of the regulatory network of the proteins sharing similar biological functions. For example, FTL (LOC_Os01g11940) encoding a florigen‐like protein and another florigen RFT1 (LOC_Os06g06300) have been reported as master regulators of rice flowering. The PPI connection between FTL and RFT1 suggests they may work as a protein complex. Moreover, FTL is also interactive with several other flowering regulators, such as OsHAP5B (LOC_Os06g45640) and OsHAPL1 (LOC_Os05g41450) in the module, implying a more complicated regulatory network of the rice flowering control (Tables S15–S18, Supporting Information).

**Figure 4:**
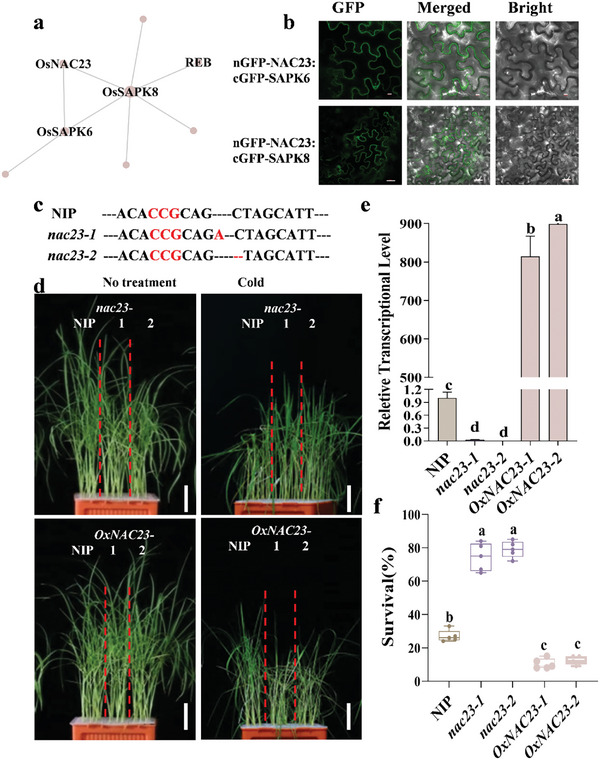
The genetic validation of *NAC23* in regulating cold stress. a) The cold stress response module shows that NAC23 interacts with abiotic stress regulators SAPK8 and SAPK6. The nodes contain genes with reported functions in cold stress response. Node size was scaled by degree score, with larger nodes representing greater scores and more importance in the network. b) BiFC assay showing the interaction of SAPK6, SAPK8 and OsNAC23 in tobacco leaf epidermal cells. Bar, 20 µm. c) Genotyping of the CRISPR/cas9 gene‐edited *nac23* mutants. A one‐bp deletion or one‐bp insertion was generated in the target site. The red highlighted letters indicate the protospacer adjacent motif (PAM) region. d) The phenotype of NIP, *nac23*, and *OxNAC23* under chilling stress (4 °C) for 5 d. *n* = 24 plants, Bar = 3 cm. e) The relative transcriptional level of *NAC23* in mutant and overexpression transgenic plants. f) The comparison of survival rates in NIP, *nac23*, and *OxNAC23* transgenic plants under chilling stress (4 °C) for 5 d. The data are presented as mean ± SD (*n* = 24 plants), Tukey's test, *α* = 0.05.

**Figure 5:**
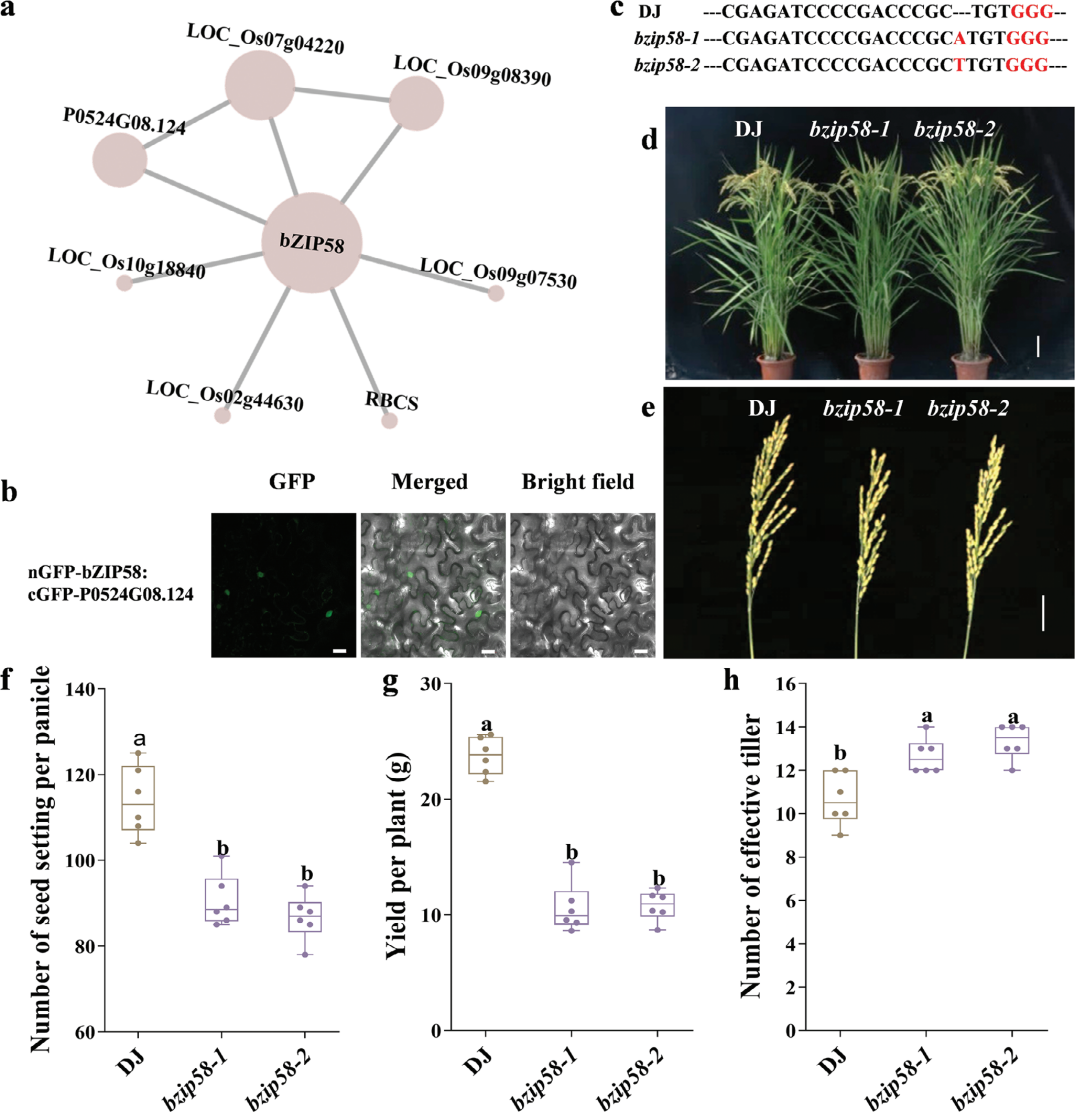
The genetic validation of *bZIP58* in regulating grain yield. a) The yield module shows that *bZIP58* interacts with grain yield‐related regulators. Node size was scaled by degree score, with larger nodes representing greater scores and more importance in the network. b) BiFC assay showing the interaction of P0524G08.124 and bZIP58 in tobacco leaf epidermal cells. Bar, 20 µm. c) Genotyping of the CRISPR/cas9 gene‐edited bzip58 mutants. A one‐bp insertion was generated in the target site of each mutant line. The red highlighted letters indicate the protospacer adjacent motif (PAM) region. d) the plant morphology of DJ and *bzip58*. Bar = 10 cm. e) Panicle morphology of DJ and *bzip58*. Bar = 3 cm. f–h) The comparison of number of seed settings per panicle, yield per plant and number of effective tillers in DJ and *bzip58*. The data are presented as mean ± SD (*n* = 6 plants), Tukey's test, *α* = 0.05.

Since most of the proteins execute their biological functions through PPI, the function of a protein could be predicted by its interactive proteins with known biological functions. In the abiotic stress‐related module, NAC23 showed PPI with cold tolerance regulators SAPK6 and SAPK8, which is further confirmed by BiFC assays (**Figure** **4**a,b). To verify the functional clues of NAC23 in cold tolerance, we generated CRISPR/Cas9‐mediated knock‐out lines and over‐expression lines of *NAC23*. Compared to the WT, *nac23* exhibited a much higher survival rate after cold treatment, whereas the over‐expression lines became more sensitive to cold stress (Figure 4c–f). In addition, we also verified the biological function of *bZIP58*, which showed PPI with several proteins in the module of yield (**Figure** **5**a). we identified bZIP58 interacting P0524G08.124 by BiFC (Figure 5b). The gene edited *bzip58* mutant had a significantly reduced seed setting per panicle than the WT, finally leading to a pronounced decrease in grain yield (Figure 5c–h).

## 4 Discussion

The significance of Protein‐Protein Interaction (PPI) analysis is widely recognized; however, PPIome profiling is still in its early stages of development. This is due to the demand for high‐throughput and accurate methodologies, which are yet to be fully established. Several reports have been applied to predict interactions according to homology‐based structure and annotation with other species.^[^ ^19^, ^20^, ^21^, ^22^, ^23^ ^]^ However, the expected PPIs typically turned out to be not high in quality. The current study developed BIP‐seq, a massive, cost‐effective, yeast two‐hybrid experiment‐based pipeline, for PPI identification. We demonstrated that BIP‐seq could finish the PPI profiling of 60 000 yeast colonies in two months with a rate of 47.5% in low‐confidence PPI and 26.3% in high‐confidence PPI, respectively, outperforming other reported PPI methods (Figure 1b and Table S16, Supporting Information). Notably, the Y2H could be extended to other two‐hybrid systems, such as bacterium two‐hybrid, mammalian membrane two‐hybrid, or Split‐Ubiquitin systems for PPI identification, or even to a Y1H system for protein‐DNA interaction profiling.^[^ ^24^, ^25^, ^26^, ^27^, ^28^, ^29^ ^]^ Beyond the application in the PPI network establishment within a particular species, BIP‐seq could also be utilized in inter‐species PPI profiling. For example, PPI information between model species and non‐model species may provide functional clues from the model species protein to its non‐model species counterparts, thus facilitating its functional genomics research. Additionally, studying the PPI between hosts and pathogens is crucial for understanding the mechanisms of pathogen infection and host response, which is essential for the prevention and treatment of diseases.

We determined to establish this method on Y2H because it is less equipment‐dependent and has an acceptable throughput compared to other methods like AP‐MS. Thus, BIP‐seq could be more affordable for most small molecular biology labs. Previously, several high through‐put, Y2H‐based PPI profiling technologies have been reported. “Stitch‐Seq” and PLATE‐seq essentially linked the bait and prey protein genes by PCR, whereas the CrY2H‐seq and RLL‐Y2H pipelines are based on the Cre recombinase interaction reporter in the yeast, which automatically fused the PPI bait and prey protein genes into one DNA fragment. As summarized in Table S16 (Supporting Information), BIP‐seq drastically outperforms the other 4 methods in PPI identification efficiency with a comparable verification rate, especially under the scenario that it was tested on cDNA libraries, which saved tremendous efforts on ORFeome construction. We attribute the higher efficiency of BIP‐seq to the combination of three simple designs, including the library versus library Y2H mating strategy, stable and mechanized dual barcode‐indexed, yeast colony PCR, and massive sequencing of DNA pools containing tens of thousands of PCR amplicons by HiFi long‐read sequencing.

Traditionally, Y2H screening has been predominantly conducted using a bait versus library mating strategy, emphasizing the importance of the target proteins. To achieve extensive interactome coverage, it is necessary to construct tens of thousands of bait vectors which require considerable effort and resources. The library versus library mating strategy allows the most effortless accessibility to tens of thousands of random PPI colonies as it skipped the most time‐consuming and costly step of large‐scale bait and prey ORFeomes construction. It is important to note that the protein‐protein interactions identified through the library versus library mating strategy are inherently random, which may result in the omission of key proteins that are of significant interest to researchers. Consequently, these two strategies should be regarded as complementary, enabling a more comprehensive coverage of the protein interactome. BIP‐seq is founded on a stable and highly efficient yeast colony PCR system, which was challenged by yeast cell wall lysis, as the lysis buffer ingredients and the released yeast cellular compounds severely inhibit the following PCR reaction. A common practice is to aliquot out a small amount of the cell lysate as a template, but it would complicate the experimental procedure and may result in poor amplifications due to the imprecise aliquot during the large‐scale PCR preparations. By adding protective additives like BSA and betaine to the cheap KOH lysis solution, we simplified the procedure to finish the 1st round of PCR reactions in a single tube and significantly reduced the cost to a shallow level. In addition, a simple, low‐cost TCS system was invented to suffice the enormous demand for thermal cyclers during BIP‐seq. Compared to the previous water bath thermal cyclers, which solely use the space on the water surface, the capacity of TCS is overwhelming because the vertical spaces of the water bath are fully used by wholly immersing the film‐sealed samples into the water. Given its high throughput and low cost, TCS could be an excellent alternative to commercial thermal cycler for small labs in developing countries. The simplified and semi‐mechanized yeast PCR pipeline enables us to finish the amplification of 10 000 Y2H colonies maximally within 5 d.

Furthermore, compared to the noise about interactions screening in liquid‐gel cultures or harvesting en masse, the picking colonies display more positive and precise interactions.^[^ ^24^, ^30^ ^]^ Finally, in contrast to the illumine sequencing used in the other 4 methods, HiFi long‐read sequencing in this study yields longer reads, which could easily cover the range of the PCR amplicons ranging from 200 to 3000 bp. It, therefore, confers more accurate identification of the PPI protein genes from their homologs or variant transcripts and the determination of the ORF fusion with the Gal4 domain.

Rice is a major crop and model species in plant molecular genetics. Although many studies have reported massively physical interactions in Arabidopsis and maize, it remains rare interactions in rice.^[^ ^31^, ^32^ ^]^ In the past decades, a couple of Y2H pairwise studies in rice have been reported with the output of a few hundred binary PPIs associated with specific functions.^[^ ^33^, ^34^, ^35^, ^36^, ^37^, ^38^ ^]^ AP‐MS and co‐fractionation mass spectrometry (CF‐MS) technologies have also been performed to detect protein complexes in rice.^[^ ^18^, ^39^, ^40^ ^]^ However, the PPIs might be bridged by other factors, thus not physical. This study created the RiPPID with 2,3032 highly‐reliable PPIs, including 22665 new PPIs, which greatly broadened the current physical PPI dataset in rice, though still far from saturating the PPIome. To the best of our knowledge, RiPPID provides the largest rice PPI network, shedding new insight into the protein domain, GO and subcellular localization preference during PPI. More importantly, the RiPPID system is capable of generating extensive modules that encompass critical biological processes such as plant height, leaf color, and heading date. These modules are a valuable resource that can be utilized to accurately predict the biological functions of the proteins within the module. In addition to the verified examples of NAC23 and bZIP58, we noticed several other genes with functional clues from their interactive proteins.

This study represents valuable broad‐scale interactions to provide linkage with dynamic and complicate functional relationships between genotype and phenotype in life.

## Conflict of Interest

The authors declare no conflict of interest.

## Author Contributions

X.L., D.X., J.L., and M.L. contributed equally to this work. J.Z. and J.W.Z. planned and designed the research. X.L., D.X., J.L., M.L., L.C., Y.C., J.H., Y.L., H.X., Y.Y., Z.L., G.L., S.W., X.Y.L., F.Z., Z.L., X.T., Y.H., Y.W., J.Y., A.M.B.U., M.A.E., and S.Z. performed experiments; X.L., D.X., and M.L. collected the raw data. J.Z., J.W.Z., X.L., D.X., M.L., W.Y., D.X., and Y.C. analyzed data; J.Z., J.W.Z., X.L., and D.X. wrote the manuscript.

## Supporting information



Supporting Information

Supporting Figure and Tables

## Data Availability

The data that support the findings of this study are available in the Supporting Information of this article.
