# Supplementary material for: Global Protein Interactome Mapping in Rice Using Barcode‐Indexed PCR Coupled with HiFi Long‐Read Sequencing
Source: Adv Sci (Weinh). 2025 Jan 22;12(11):2416243. doi: 10.1002/advs.202416243 (PMC11923860; doi:10.1002/advs.202416243)
Supplement: Supplementary file 1 — Supporting Information [file ADVS-12-2416243-s002.docx]

Supporting Information

**Global Protein Interactome Mapping in Rice Using Barcode-indexed PCR Coupled with HiFi Long-read Sequencing**

Xixi Liu^#^, Dandan Xia^#^, Jinjin Luo^#^, Mengyuan Li^#^, Lijuan Chen, Yiting Chen, Jie Huang, Yanan Li, Huayu Xu, Yang Yuan, Yu Cheng, Zhiyong Li, Guanghao Li, Shiyi Wang, Xinyong Liu, Wanning Liu, Fengyong Zhang, Zhichao Liu, Xiaohong Tong, Yuxuan Hou, Yifeng Wang, Jiezheng Ying, Abdullaev Mirtemir Baxodir ugli, Mukhammadjon Arabboevich Ergashev, Sanqiang Zhang, Wenya Yuan, Dawei Xue, Jianwei Zhang^*^ and Jian Zhang^*^


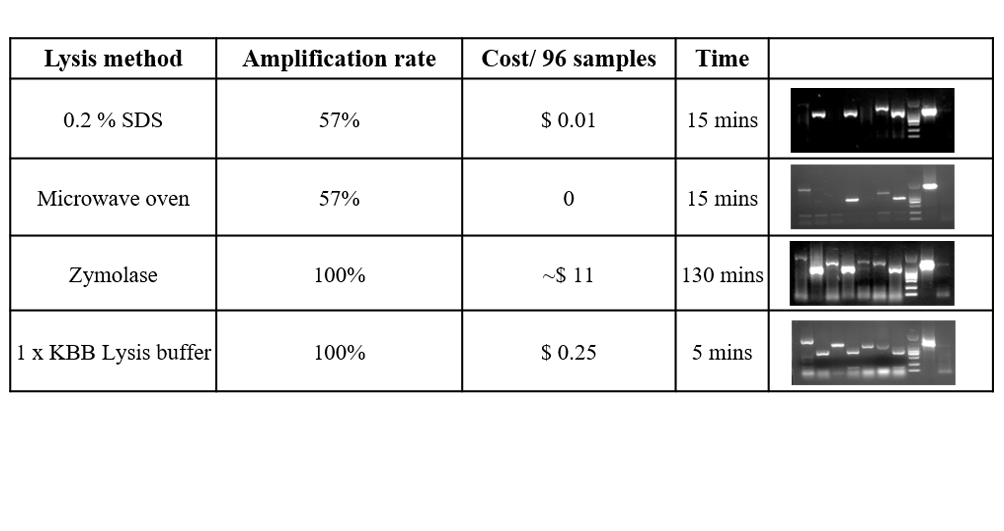


**Figure S1.** The amplification rate, cost, and time comparison for yeast colony lysis using KBB Lysis buffer and other traditional methods. The cost was estimated based on the market prices in China.


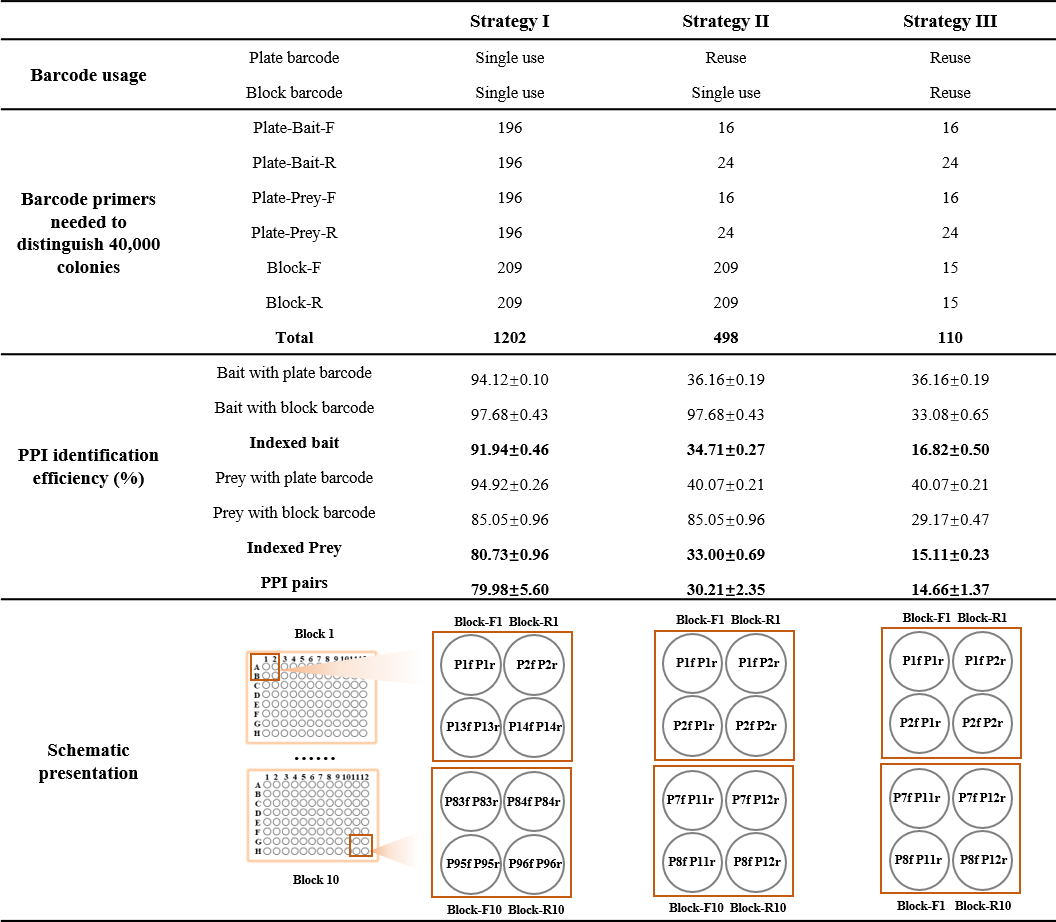


**Figure S2.** The comparison of PPI identification efficiency using three barcode design strategies. To identify the efficiency of PPI, we used three strategies for barcode usage during barcode-indexed PCR. Strategy I: The block and plate barcode primers are used singly. Strategy II: The block barcode primers are used singly, while the plate barcode primers are reused. Strategy III: The block and plate barcode primers are reused.


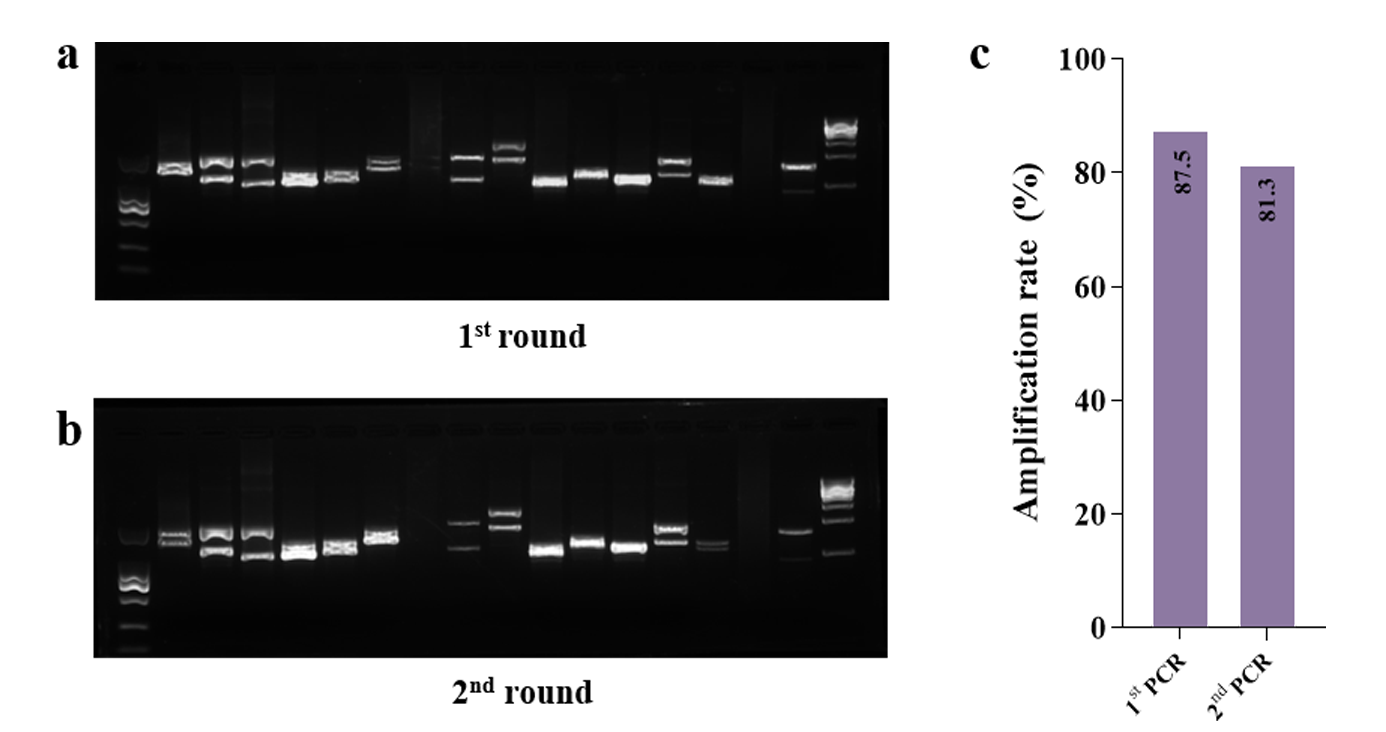


**Figure S3.** The amplification rate from yeast colony with barcode-indexed PCR method. a) The agarose electrophoresis of the first round PCR products. b) The agarose electrophoresis of the first round PCR products. c) The statistics of the PCR amplification rate.


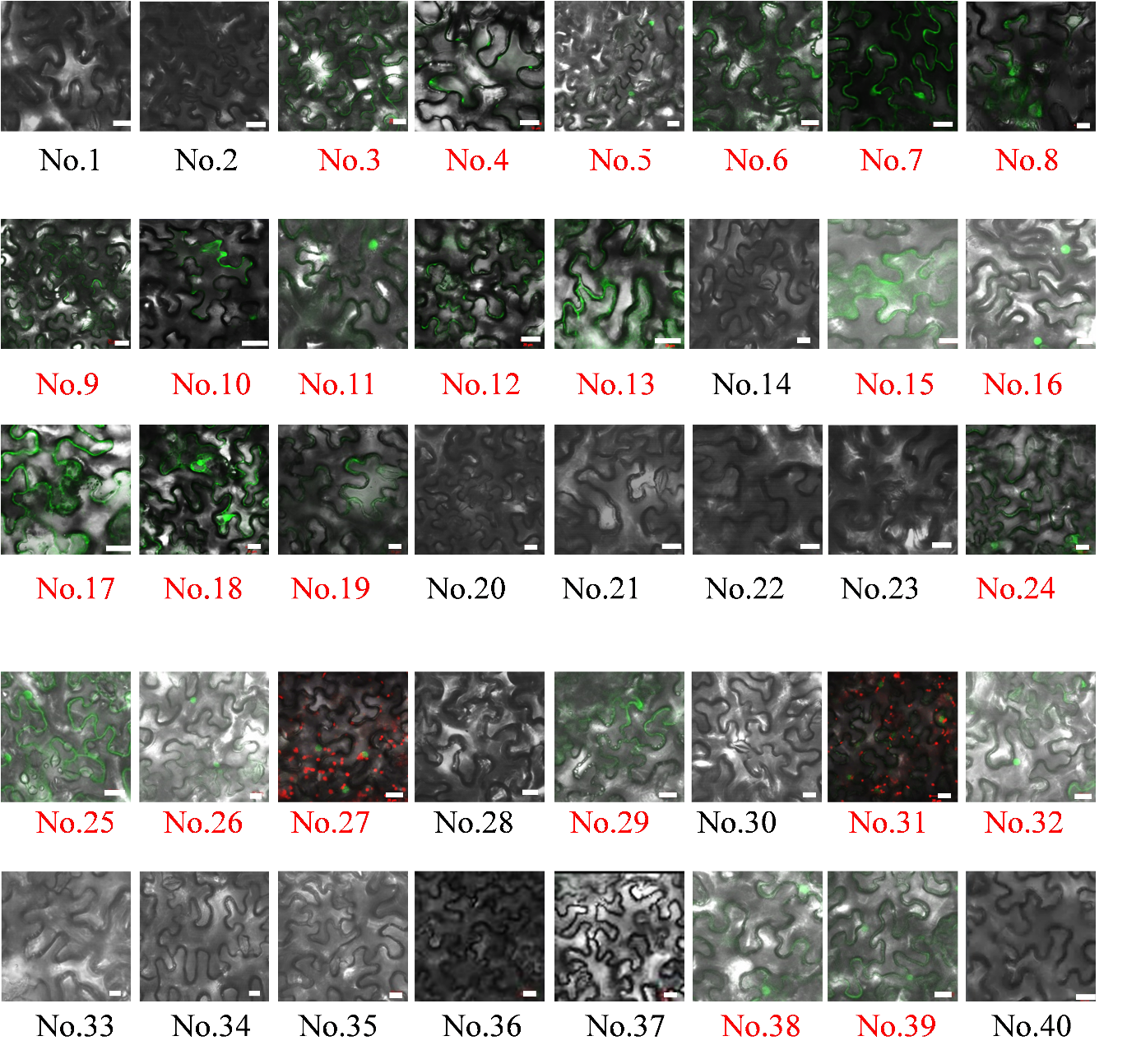


**Figure S4 |** Verification of 40 randomly selected PPIs in RiPPID using BiFC. The red font indicates the positive PPI retested by BiFC. Bars indicates 20 µm. All the retested protein IDs can be found in Table S10.


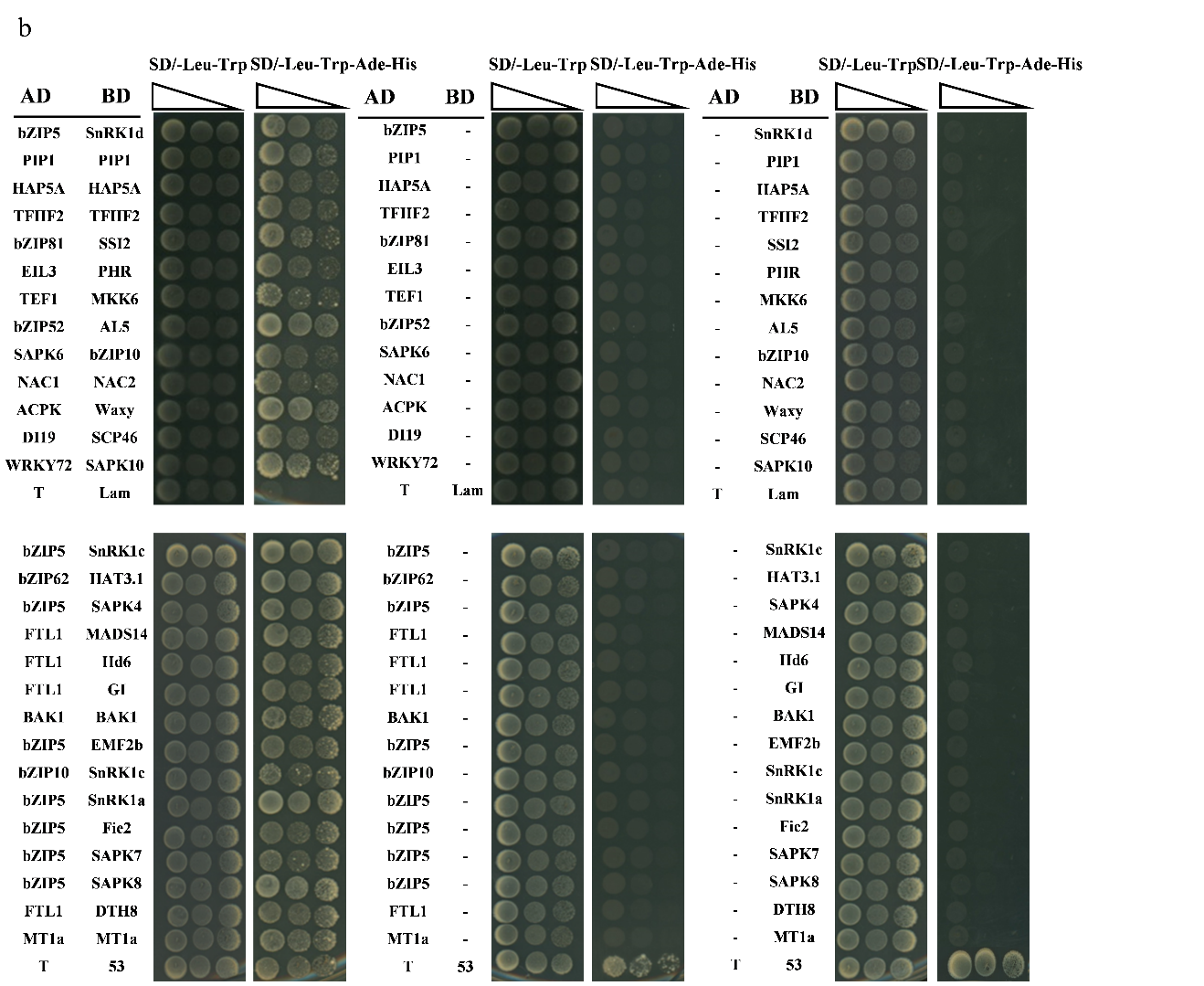

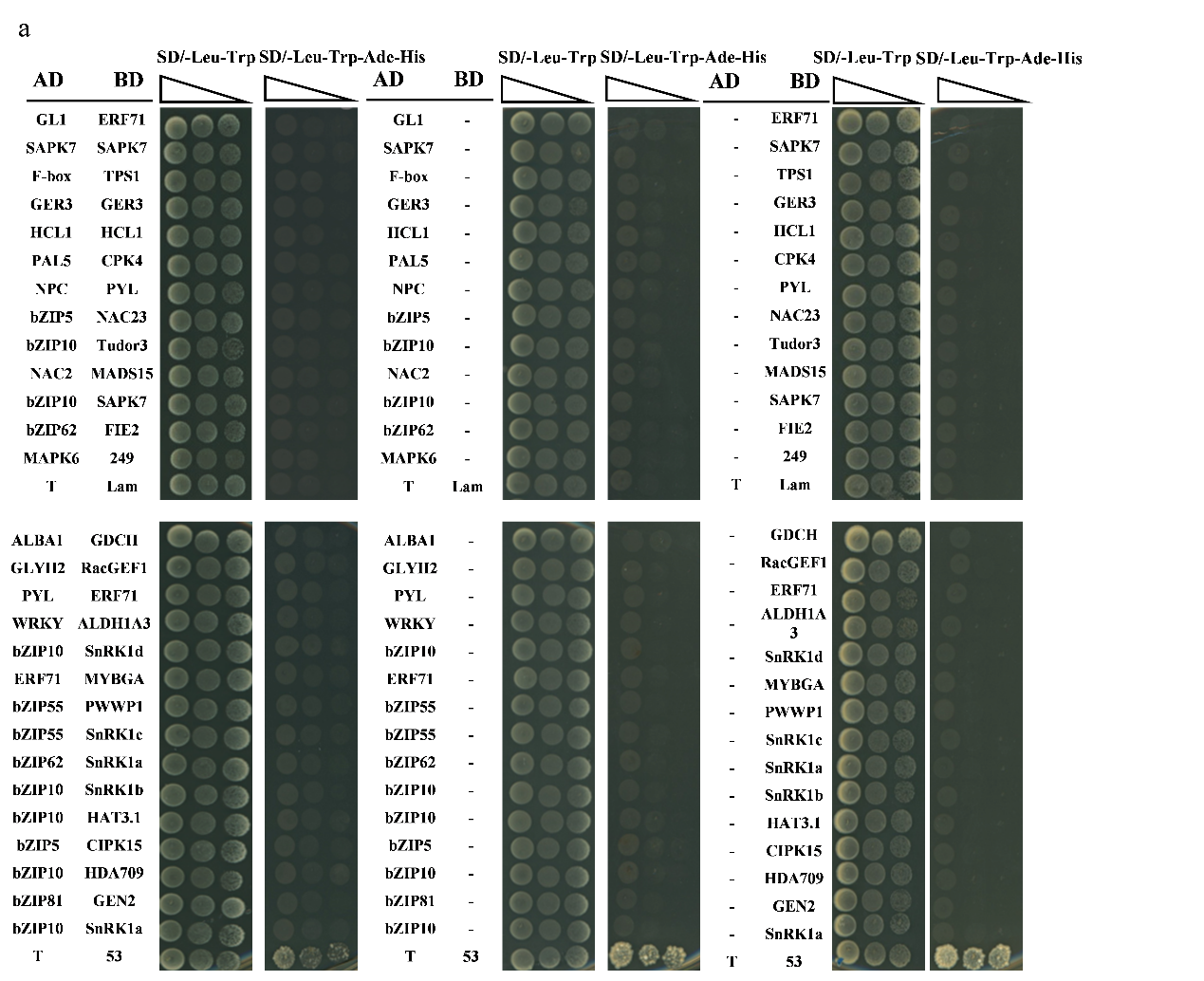


**Figure S5.** (a-b) Y2H analysis of 28 negative and 28 positive PPIs, respectively. AD, pGADT7; BD, pGBKT7; pGADT7-T and pGBKT7-53 were used as positive controls, while pGADT7-T and pGBKT7-Rec were used as negative controls.


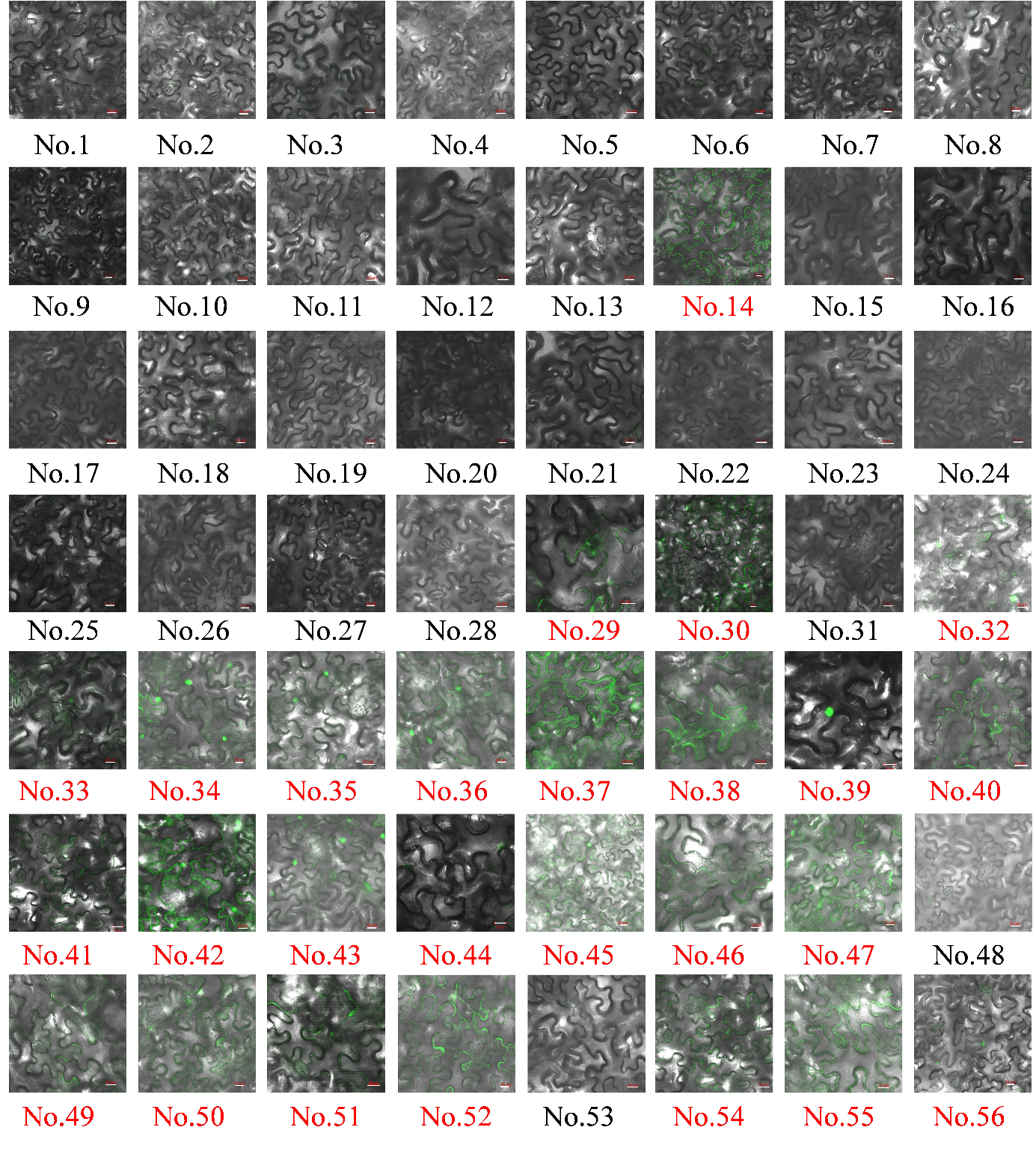


**Figure S6.** Validation of the positive and negative Y2H PPIs by BiFC. Bars indicates 20 µm.

**
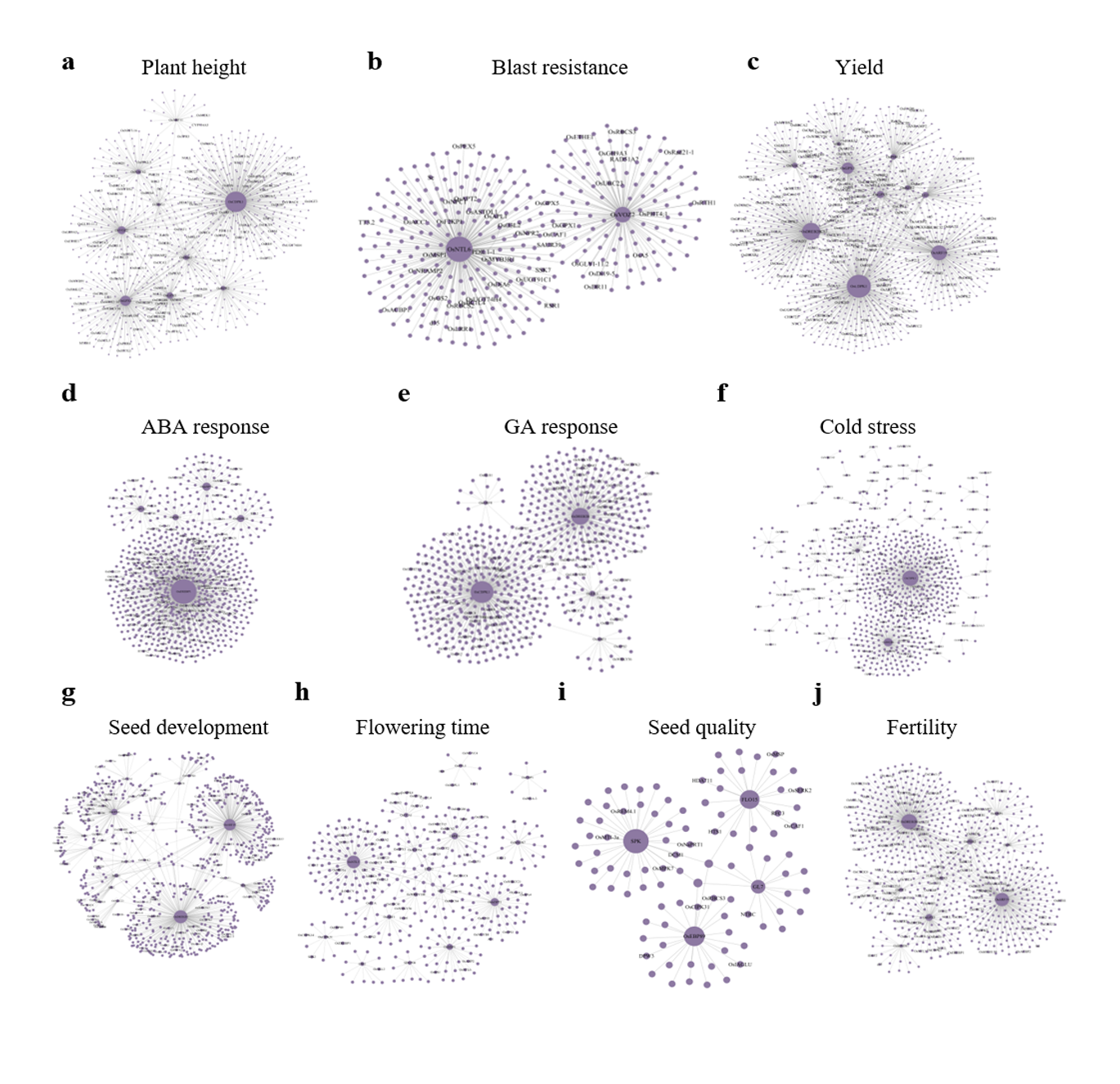
Figure S7.** The ten functional modules are classified by trait ontology (TO) in RiPPID. a) plant height subnetwork b) blast resistance subnetwork c) yield subnetwork d-e) ABA and GA response subnetwork f) cold stress subnetwork g) seed development subnetwork h) flowering time subnetwork i) seed quality subnetwork j) fertility subnetwork. The node size represents degree scores, with larger nodes representing greater scores and greater importance in the subnetwork.

**Supporting information**

Additional supporting information may be found online in supporting information section at the end of the article.

**Supplementary Table:**

Supplementary Table 1. The 1736 auto-activations PPIs identified in this study

Supplementary Table 2. The 23,032 PPIs identified in this study

Supplementary Table 3. Comparison of RiPPID with previously reported dataset

Supplementary Table 4. The detailed information of homodimers

Supplementary Table 5. Cellular localization of the detected PPI proteins

Supplementary Table 6. GO categories of the detected PPI proteins

Supplementary Table 7. Counts of GO categories of the PPI proteins

Supplementary Table 8. Domains counts in PPIs (count >6)

Supplementary Table 9. PPIs containing kinases and TFs

Supplementary Table 10. Counts of PPIs between kinases and TFs

Supplementary Table 11. BiFC verification of PPIs

Supplementary Table 12. The validation of PPIs in Y2H and BiFC asasy

Supplementary Table 13. The false-positive rate and false-negative rate of BiFC

Supplementary Table 14. Nodes and modules of the PPIs (class>5)

Supplementary Table 15. Trait ontology analysis of PPIs involved in various pathways

Supplementary Table 16. Comparison of the reported PPIome profiling methods

Supplementary Table 17. Sequences of primers used in this study

Supplementary Table 18. Sequences of barcode used in this study

**Supplementary File:**

Supplementary File 1. Demonstration of PCR using thermal cycling station (TCS)
