# Supplementary figures and images for: Global Protein Interactome Mapping in Rice Using Barcode‐Indexed PCR Coupled with HiFi Long‐Read Sequencing
Source: Adv Sci (Weinh). 2025 Jan 22;12(11):2416243. doi: 10.1002/advs.202416243 (PMC11923860; doi:10.1002/advs.202416243)

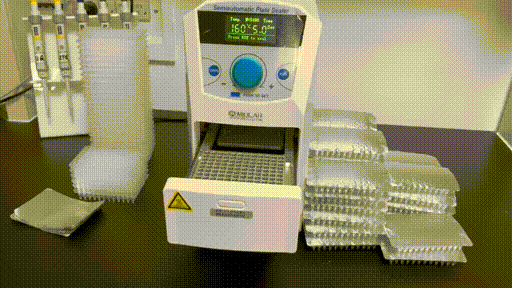

Supplement: Supplementary file 2 — Supporting Figure and Tables [file ADVS-12-2416243-s001.zip › advs202416243-sup-0002-SuppFigTables/Supplemental file 1.gif]
